# Quantitative bone single-photon emission computed tomography imaging for uninfected nonunion: comparison of hypertrophic nonunion and non-hypertrophic nonunion

**DOI:** 10.1186/s13018-021-02279-8

**Published:** 2021-02-10

**Authors:** Keisuke Oe, Feibi Zeng, Tomoaki Fukui, Munenobu Nogami, Takamichi Murakami, Tomoyuki Matsumoto, Ryosuke Kuroda, Takahiro Niikura

**Affiliations:** 1 Department of Orthopaedic Surgery, Kobe University Graduate School of Medicine, 7-5-1 Kusunoki-cho, Chuo-ku, Kobe, 650-0017 Japan; 2 Department of Radiology, Kobe University Graduate School of Medicine, 7-5-1 Kusunoki-cho, Chuo-ku, Kobe, 650-0017 Japan

**Keywords:** Bone SPECT, SUV, Hypertrophic nonunion, Non-hypertrophic nonunion, Autologous bone graft

## Abstract

**Background:**

Recently, a standardized uptake value (SUV) has been used to evaluate bone single-photon emission computed tomography (SPECT). The aim of this study was to investigate quantitative SPECT imaging of uninfected nonunion to compare hypertrophic nonunion and non-hypertrophic nonunion using volume-based parameters.

**Methods:**

We evaluated 23 patients with uninfected nonunion who underwent SPECT acquisition 3 h after an injection of ^99m^Tc-hydroxymethylene diphosphonate or ^99m^Tc-methylene diphosphonate from April 2014 to November 2019. We reconstructed the acquired data and performed voxel-based quantitative analysis using the GI-BONE software. Quantitative parameters, maximum SUV (SUV_max_), peak SUV (SUV_peak_), and mean SUV (SUV_mean_) in the high and low uptake areas of nonunion were compared between hypertrophic nonunion and non-hypertrophic nonunion. The contralateral limb was used as a control, and the ratios of the quantitative parameters were calculated.

**Results:**

The values for the quantitative parameters (high uptake area/low uptake area, respectively), SUV_max_ control ratio (12.13 ± 4.95/6.44 ± 4.71), SUV_peak_ control ratio (11.65 ± 4.58/6.45 ± 4.64), and SUV_mean_ control ratio (11.94 ± 5.03/6.28 ± 4.95) for hypertrophic nonunion were higher than those for non-hypertrophic nonunion (7.82 ± 4.76/3.41 ± 2.09 (*p* = 0.065/0.12), 7.56 ± 4.51/3.61 ± 2.23 (*p* = 0.065/0.22), and 7.59 ± 5.18/3.05 ± 1.91 (*p* = 0.076/0.23)).

**Conclusions:**

SUV_max_, SUV_peak_, and SUV_mean_ control ratios obtained from bone SPECT images can quantitatively evaluate the biological activity of nonunions and may be an effective evaluation method for treatment decisions, especially the necessity of autologous bone grafting.

## Background

Nonunion occurs in approximately 5% of all fractures [1–3]. The basis of treatment of nonunion is to fully understand and address the causes. There are various causes of nonunion, and these are largely divided into biological factors and mechanical factors [4–7]. Generally, the cause of nonunion is determined by X-ray findings, with the presence of marked callus formation around the nonunion site considered to indicate biological activity. In cases of nonunion without callus formation, it is very difficult to determine the existence of biological activity using only X-ray findings. In some cases, biological activity exists around the nonunion without callus in X-ray findings. Bone scintigraphy is considered an important examination for determining biological activity [8–12]. Uptake in bone scintigraphy reflects blood flow and new bone formation [13, 14]. Although bone scintigraphy is excellent for qualifying biological activity, it is difficult to quantitatively evaluate biological activity using scintigraphy.

Recently, a standardized uptake value (SUV) has been applied to evaluate bone single-photon emission computed tomography (SPECT) [15–18]. However, to the best of our knowledge, no published report has discussed SUV measurement in nonunion imaging using SPECT scans with ^99m^Tc hydroxymethylene diphosphonate (^99m^Tc-HMDP) or ^99m^Tc methylene diphosphonate (^99m^Tc-MDP). The aim of this study was to investigate quantitative SPECT imaging for uninfected nonunion and to compare hypertrophic nonunion and non-hypertrophic nonunion using volume-based parameters.

## Materials and methods

### Patients

Our institutional review board approved this retrospective study and waived the requirement for informed patient consent. Twenty-three of the 52 patients who underwent bone SPECT at our institution from April 2014 to November 2019 had uninfected nonunion of the femur, tibia, or humerus. Exclusion criteria were infectious diseases (osteomyelitis, purulent arthritis); nonunion cases with pelvic, fibular, clavicular, radial, and atypical femoral fractures; contralateral fracture cases; bone tumor cases (metastatic and primary); and unknown patient height and weight.

Patients’ medical records were evaluated to determine their characteristics and treatment progress. The average age at the time of bone SPECT scan was 43.8 ± 18.1 years (range, 16–76 years), with 13 men and 10 women. There were 15 femoral nonunions, 5 tibial nonunions, and 3 humeral nonunions. The implant types were intramedullary nails in 15 cases, plates in 6 cases, hemiarthroplasty + plate in 1 case, and no implant in 1 case. The average period from the injury to bone SPECT scan was 574 ± 854.3 days (range, 89–4330 days). The number of operations before the SPECT scan was 1.8 (1 in 11 cases, 2 in 6 cases, 3 in 5 cases, and conservative treatment in 1 case). Bone-modifying agents (teriparatide acetate) for osteoporosis treatment were used in four cases. Low-intensity pulsed ultrasound treatment was used in 16 cases, and no cases received steroid medication. Autologous bone grafts were performed in 13 cases. Bone union was achieved in all cases after the operations following the SPECT scans.

There were 8 hypertrophic nonunions and 15 non-hypertrophic nonunions. We defined hypertrophic nonunion as elephant foot and horse hoof, and non-hypertrophic nonunion as oligotrophic, comminuted (torsion-wedge, dystrophic, necrotic), defect, and atrophic, according to the Weber classification of X-ray findings [9, 19]. Three senior orthopedic trauma surgeons classified the nonunions; 19/23 cases had the same classification by all three surgeons, but 4 cases had different opinions, so a consensus meeting was held, and the opinions were unified.

### SPECT study

SPECT scans were performed before nonunion surgery. ^99m^Tc-MDP or ^99m^Tc-HMDP was injected intravenously, and SPECT imaging was performed 3 h later. SPECT scans were obtained using a SPECT scanner (E.CAM; Canon Medical Systems Corp., Tokyo, Japan). The SPECT scan was acquired using a low-energy, high-resolution collimator at 140 keV photoenergy peak for ^99m^Tc with a 128 × 128 matrix of 4.8-mm pixel size, and a total of 60 projections (30 steps) over 360° with a dwell time of 10 s/step. SPECT images were reconstructed using three-dimensional-ordered subset expectation maximization (3D-OSEM) with six iterations, 15 subsets, and a Butterworth filter.

### SUV measurements

The quantitative SPECT parameters were calculated using the software, GI-BONE (AZE, Tokyo, Japan). The SUV was calculated for the quantitative analysis of ^99m^Tc-MDP or ^99m^Tc-HMDP uptake, as follows:

SUV = (tissue radioactivity/voxel volume)/(injected radioactivity/body weight), where tissue radioactivity means a tissue radioactivity concentration measured by SPECT. Tissue radioactivity concentration was obtained by multiplying the SPECT counts and Becquerel calibration factor, which was determined by scanning the cylindroid phantom filled with a known radioactivity concentration. Various SUV parameters were calculated using GI-BONE. The maximum value for SUV (SUV_max_) = (maximum radioactivity/voxel volume)/(injected radioactivity/body weight). The mean value for SUV (SUV_mean_) = (total radioactivity/volume of interest (VOI))/(injected radioactivity/body weight). The peak value of SUV (SUV_peak_) represents the average SUV obtained within a 1-cm^3^ sphere of the region of interest centered on the highest voxel of the target area.

The VOI size was defined as a sphere with a diameter of 19.2-mm sphere, considering the limit of spatial resolution of SPECT [20]. Three parts of the VOI were placed on the healthy opposite extremity, and the average value was used as the control value; the SUV control ratio of the nonunion was used for evaluation. When comparing between patients, we considered that it is desirable to evaluate not by the absolute value of the target site but by the control ratio. The orthopedic trauma surgeon and the radiologist identified the nonunion site by observing plain X-ray, computed tomography (CT), and SPECT images, and identified and measured the hot and cold uptake areas.

### Statistical analysis

Statistical analyses for the SPECT parameters of the nonunion lesions (hypertrophic nonunion and non-hypertrophic nonunion) were performed using the Mann–Whitney *U* test. All statistical analyses were performed using GraphPad Prism 7 (GraphPad Software Inc., La Jolla, CA). A *p* value less than 0.05 was considered statistically significant.

## Results

The characteristics of the 23 patients and the SPECT findings for nonunion lesions using volume-based parameters are shown in Table 1.

**Table 1:** Characteristics of the 23 patients and the SPECT findings for nonunion lesions using volume-based parameters

Case	Age	Gender	Lesion	Classification	Control			Control ratio (high uptake area/low uptake area)		
					SUV_max_	SUV_peak_	SUV_mean_	SUV_max_	SUV_peak_	SUV_mean_
1	37	M	Tibia	Hypertrophic	1.2	1.1	0.8	19.1/10.2	17.9/10.8	20.4/12.3
2	27	F	Femur	Hypertrophic	1.2	1.1	0.9	15.3/15.3	14.9/14.9	14.3/14.3
3	29	M	Femur	Hypertrophic	1.8	1.7	1.6	11.3/9.6	11.1/9.5	9.8/9.5
4	16	M	Femur	Hypertrophic	2.1	1.9	1.6	15.6/3.3	14.9/3.3	14.7/2.9
5	59	F	Femur	Hypertrophic	3.7	3.5	3.4	3.7/2.6	3.6/2.7	3.4/2.0
6	45	M	Femur	Hypertrophic	1.8	1.7	1.4	8.4/2.5	8.0/2.6	9.0/2.6
7	17	M	Femur	Hypertrophic	1.1	1.0	0.8	9.1/4.1	9.2/4.0	10.2/3.0
8	70	F	Femur	Hypertrophic	2.1	1.9	1.7	14.5/3.9	13.5/3.9	13.7/3.6
9	74	F	Humerus	Non-hypertrophic	1.4	1.2	1.1	3.1/0.5	3.3/0.5	1.5/0.4
10	24	M	Humerus	Non-hypertrophic	2.0	1.9	1.7	6.2/5.3	5.9/5.2	3.9/3.6
11	48	F	Tibia	Non-hypertrophic	1.6	1.5	1.3	3.5/3.5	3.5/3.5	3.6/3.6
12	21	M	Femur	Non-hypertrophic	1.0	0.9	0.8	6.7/3.1	6.8/3.4	6.4/2.7
13	40	M	Tibia	Non-hypertrophic	1.0	0.9	0.8	12.1/5.4	11.7/5.6	10.7/4.0
14	76	F	Humerus	Non-hypertrophic	2.1	1.9	1.8	3.7/3.7	3.8/3.8	3.6/3.4
15	68	F	Tibia	Non-hypertrophic	3.0	2.8	2.4	8.0/3.2	7.7/3.7	9.1/3.4
16	49	F	Tibia	Non-hypertrophic	1.9	1.8	1.5	15.8/3.5	14.9/3.6	14.9/2.9
17	47	M	Femur	Non-hypertrophic	1.5	1.4	1.2	10.3/3.0	9.5/3.5	8.0/2.1
18	59	F	Femur	Non-hypertrophic	4.6	4.3	3.7	4.9/1.4	4.7/1.7	5.0/1.2
19	38	M	Femur	Non-hypertrophic	3.2	3.0	2.6	2.2/0.5	2.5/0.5	2.5/0.5
20	54	F	Femur	Non-hypertrophic	3.6	3.3	3.1	4.6/2.5	4.4/2.5	4.0/2.2
21	33	M	Femur	Non-hypertrophic	1.7	1.7	1.3	10.4/2.6	10.0/2.7	12.2/2.9
22	54	M	Femur	Non-hypertrophic	1.4	1.3	1.1	7.3/3.9	6.8/4.2	7.9/4.5
23	23	M	Femur	Non-hypertrophic	1.4	1.3	1.1	18.4/8.9	17.9/9.7	20.2/8.4

*SPECT* single-photon emission computed tomography, *SUV* standardized uptake value, *M* male, *F* female

Table 2 shows the quantitative SPECT parameters for hypertrophic nonunion and non-hypertrophic nonunion (high uptake area of the nonunion lesion data/low uptake area of the nonunion lesion data). The SUV_max_ control ratio (12.13 ± 4.95/6.44 ± 4.71), SUV_peak_ control ratio (11.65 ± 4.58/6.45 ± 4.64), and SUV_mean_ control ratio (11.94 ± 5.03/6.28 ± 4.95) for the hypertrophic nonunions were higher than those for the non-hypertrophic nonunions [7.82 ± 4.76/3.41 ± 2.09 (*p* = 0.065/0.12), 7.56 ± 4.51/3.61 ± 2.23 (*p* = 0.065/0.22), and 7.59 ± 5.18/3.05 ± 1.91 (*p* = 0.076/0.23), respectively].

**Table 2:** Quantitative SPECT parameters for hypertrophic nonunion and non-hypertrophic nonunion

Parameters	Hypertrophic nonunion	Non-hypertrophic nonunion	*p* value
SUV_max_ control ratio	12.13 ± 4.95/6.44 ± 4.71	7.82 ± 4.76/3.41 ± 2.09	0.065/0.12
SUV_peak_ control ratio	11.65 ± 4.58/6.45 ± 4.64	7.56 ± 4.51/3.61 ± 2.23	0.065/0.22
SUV_mean_ control ratio	11.94 ± 5.03/6.28 ± 4.95	7.59 ± 5.18/3.05 ± 1.91	0.076/0.23
Age	37.5 ± 19.42	47.2 ± 17.66	0.22

The high uptake area data appear before the forward slash, and the low uptake area data appear after the forward slash

*SUV* standardized uptake value

Analysis of the low uptake areas revealed two cases with lower values than the control (case 9 and case 19 in Table 1).

### Case presentation (case 19)

**Fig. 1:**
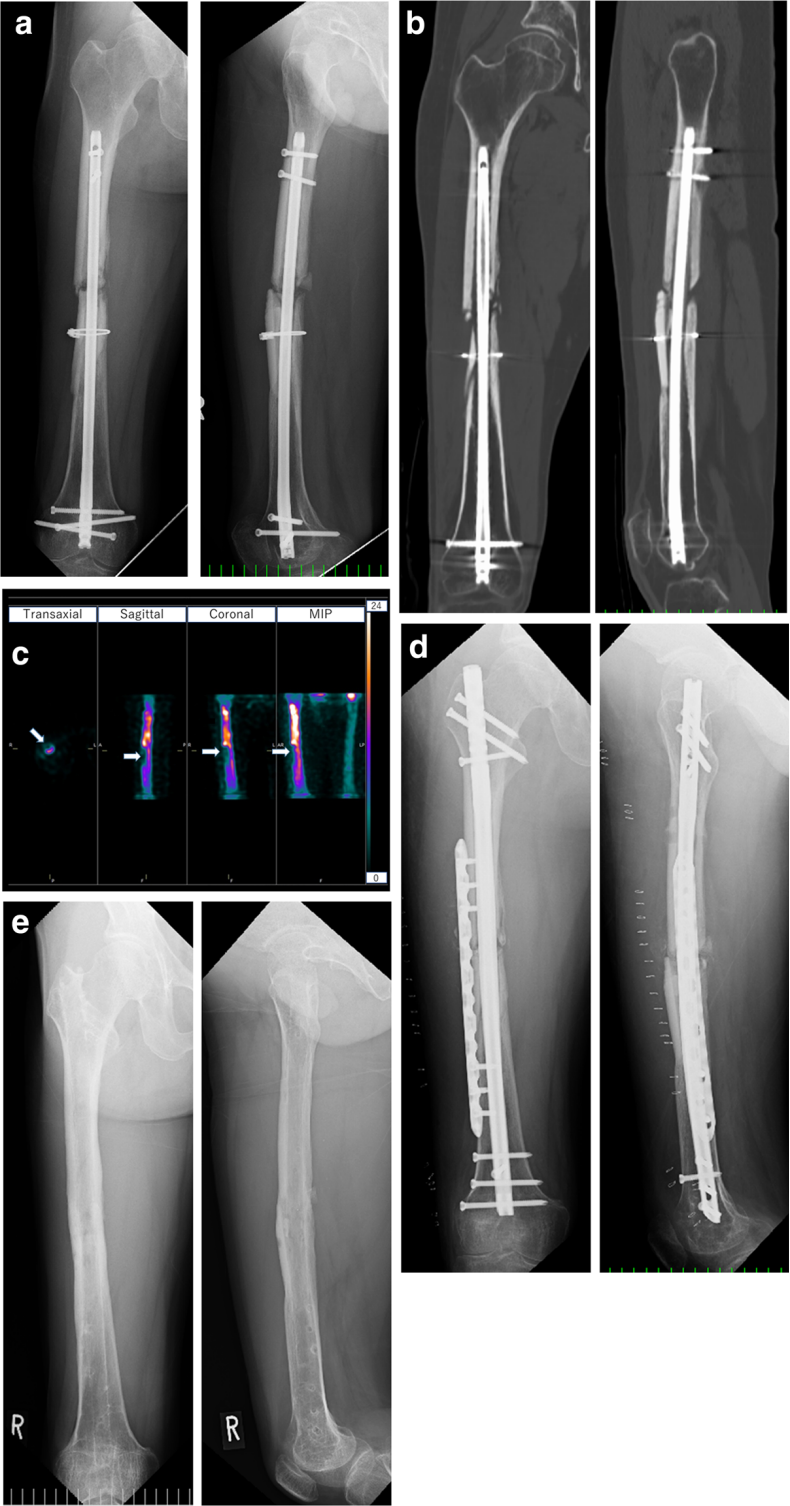
Case 19. A 38-year-old man with noninfected nonunion of the femur. **a** X-ray image at the time of admission to our hospital. **b** CT images from the same period. A relatively large bone gap was found around the nonunion site. **c** Quantitative evaluation by the GI-BONE software. The third bone fragment shows low uptake (white arrow). MIP, maximum intensity projection. **d** X-ray image immediately after nonunion surgery with augmentative plating, nail exchange, and autologous bone grafting. **e** X-ray image 6 months postoperatively showing that bone union was achieved. All implants were removed

The patient was a 38-year-old man who sustained a closed right femoral shaft fracture in a traffic accident. The femoral shaft fracture with a relatively large third bone fragment was fixed with an intramedullary retrograde nail and cable wire at another hospital. One year later, he was referred to our hospital for treatment of nonunion, and a relatively large bone gap was found around the nonunion site (Fig. 1a and b). Visually, uptake in the third bone fragment was very low (Fig. 1c). The SUV of the proximal to the nonunion area was higher than the control, but the SUV of the third bone fragment distal to the nonunion area was lower than the control (the SUV_max_ control ratio, SUV_mean_ control ratio, and SUV_peak_ control ratio in this area were 0.5, 0.5, and 0.5, respectively) (Table 1). The patient underwent nail exchange, augmentative plating, and autologous bone grafting harvested using the reamer-irrigator-aspirator (RIA) system and achieved bone union 6 months after the operation (Fig. 1d and e).

## Discussion

There have been reports of qualitative evaluation of nonunion by bone scintigraphy [9, 10], but to our knowledge, ours is the first report to quantify bone SPECT in nonunion. We found that SUV can be quantified from bone SPECT at the nonunion site. Our results after performing SUV quantification of bone SPECT showed no statistically significant difference between the hypertrophic nonunion group and the non-hypertrophic nonunion group. The reason for this finding was that some cases of non-hypertrophic nonunion had a high SUV control ratio; however, few cases of hypertrophic nonunion had a low SUV control ratio. This is an important finding that numerically suggests that it is not possible to completely grasp the biological activity using only X-ray images of the morphology of the nonunion.

Quantification allows comparison with a healthy part (control). It is very important to compare the SUV of the control and the nonunion because there are individual differences in the accumulated values in the control (Table 1) [15]. Autologous bone grafts are necessary for treatment because biological activity is not sufficient if the SUV of the nonunion site is lower than that of the control. Quantitative SPECT parameters, such as SUV_max_, SUV_peak_, and SUV_mean_, could be useful tools to evaluate biological activity at the nonunion site. Previous reports state that SUV_peak_ is especially useful among these parameters; however, we found a similar tendency for all parameters, in this study. We consider the reason why we found no difference with each parameter is that the VOI size was unified to a 19.2-mm sphere in all cases, whereas many studies measured the VOI size over a wide range [15–18].

**Fig. 2:**
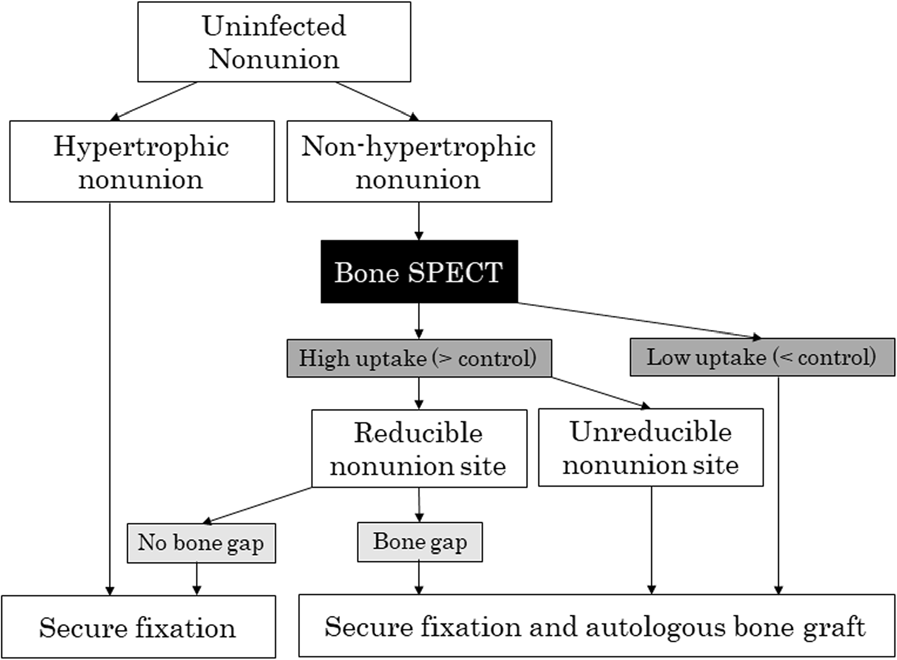
Algorithm of uninfected nonunion treatment

The authors evaluated a treatment algorithm using bone SPECT in uninfected nonunion. All cases of hypertrophic nonunion showed higher values than the control. These cases do not need bone SPECT examination; the treatment is to obtain proper fixation. Even in cases of non-hypertrophic nonunion, if both the high uptake area and the low uptake area have higher SUV values than the control, good reduction at the nonunion site is achieved, and no bone gap is observed, autologous bone grafting is not performed; only secure fixation is performed. However, if a bone gap is recognized even if reduction is achieved, secure fixation and autologous bone grafting are necessary. In contrast, if the reduction procedure results in unacceptable reduction or poor joint compatibility, secure fixation and autologous bone grafting are required, which is also required if the SUV value is lower than the control (Fig. 2). Types of autologous bone include iliac bone and RIA bone. These are very useful for obtaining biological activity, but there are reports of complications such as pain, fractures, bleeding, hematoma, infection, and nerve palsy around the donor site [21–23]. These complications might be avoided by performing bone SPECT examination and appropriately determining the indication for autologous bone graft.

There are limitations in this study. First, the spatial resolution of SPECT is relatively low. Generally, the reliability of bone SPECT is not high when quantifying a target of ≤ 17 mm, so there is room for further study in cases of small bone nonunion such as the hand, foot, forearm, and clavicle. Second, attenuation correction of gamma rays was not performed because there was no CT that can be used for attenuation correction. Additionally, several issues can attenuate gamma rays, such as the type of metal implant, and this warrants further investigation. We have not been able to correct attenuation in this study, but we would like to evaluate CT at the same time as SPECT and the quantitative value of attenuation correction using CT in the future.

## Conclusion

SUV_max_, SUV_peak_, and SUV_mean_ control ratios obtained from bone SPECT images can quantitatively evaluate the biological activity of a nonunion site and might be an effective evaluation tool for treatment decisions, especially regarding the necessity of autologous bone grafting.

## Data Availability

The datasets during and/or analyzed during the current study are available from the corresponding author on reasonable request.
